# Berg on Belief Reports

**DOI:** 10.1007/s11406-016-9755-2

**Published:** 2016-09-05

**Authors:** Anthony Everett

**Affiliations:** Department of Philosophy, University of Bristol, Cotham House, Cotham Hill, Bristol, BS6 6JL UK

**Keywords:** Belief-reports, Millianism, Frege-cases, Conversational implicature

## Abstract

Jonathan Berg’s insightful and lucid book Direct Belief develops a pragmatic account of our intuitions about Frege-cases. More precisely Berg argues that our practice of belief-reporting normally exhibits certain regularities. He argues that utterances of belief reports typically conversationally implicate that the reports adhere to these regularities. And he uses these implicatures to explain our intuitions about Frege-cases. I explore and unpack Berg’s pragmatic account, considering and offering responses to three natural worries that might be raised. In particular, I respond to the objection that the regularities Berg invokes cannot generate the conversational implicatures he claims. I respond to the objection that the regularities Berg invokes do not, in fact, obtain. And I respond to the worry that Berg cannot explain how these regularities might arise in the first place.

Jonathan Berg’s insightful and lucid book *Direct Belief* (Berg 2012) has two central and related aims, although it does much more besides along the way. The first of these aims is to argue for and develop a pragmatic account of our intuitions about Frege-cases, invoking the phenomenon of conversational implicature to explain why, for example, in certain contexts we are inclined to judge utterance of:

1. Lois Lane believes that Superman can fly,
  as true but utterances of:
2. Lois Lane believes that Clark Kent can fly,
  as false. The second of these aims is, as the title suggests, is to argue that belief is a direct, unmediated, relation between the believer and the proposition believed. I have a great deal of sympathy for Berg’s project and I will not take substantial issue with his conclusions. In what follows, however, I want to explore and unpack Berg’s pragmatic account of our intuitions concerning Frege-cases, in part by considering and offering responses to some natural objections that might be raised against it. In doing so I will suggest some friendly amendments to Berg’s account. I am not sure whether Berg would agree with these amendments or with everything I have to say. But I offer what follows in the spirit of a Millian fellow-traveler.

## Berg’s Account

As noted, Berg argues that we should explain our sense that, at least in some contexts, utterances of (1) are true while utterances of (2) are false in terms of conversational implicature. For Millians such as Berg, utterances of (1) and (2) have the same semantic content. They both express a Russellian proposition that we can represent as <Lois, belief, <Superman, can fly>>. However Berg suggests that utterances of (1) will standardly generate different conversational implicatures from utterances of (2). In certain contexts the former implicatures will be true while the latter will be false. And it is this that explains our intuition that utterances of (1) in those context are true and utterances of (2) false.

This sort of pragmatic explanation of our intuitions about such cases is, of course, not new to Berg. However Berg’s account is the most nuanced and detailed version of the pragmatic approach of which I am aware. It is one thing to maintain that utterances of (1) and (2) generate different conversational implicatures of a certain form. It is another to give a plausible account of how, precisely, these implicatures are generated. The devil is in the details here and Berg’s account is commendably detailed. And before proceeding I want to note two further attractive aspects of Berg’s account.

Firstly, Berg’s account provides a nice systematic and uniform account of how the implicatures driving our intuitions in Frege-cases are generated. Nevertheless, as we will see, it allows for the implicatures driving our intuitions in different Frege-cases to have different subject matters. To see why this is an attractive feature of Berg’s account, consider the following two cases. Firstly, to take an example from Berg, consider Rover who wags his tail when he sees Superman in his superhero costume but snarls and growls when he sees Superman in his Clark-Kent outfit. In these circumstances it is natural to hear utterances of (3) as true and (4) as false:

(3) Rover believes that Superman is his friend,
(4) Rover believes that Clark Kent is his friend.

If we want to explain our intuitions in terms of utterances of (3) and (4) generating different conversational implicatures the question arises as to precisely what these implicatures might be. Perhaps we might take these implicatures to attribute certain (non-linguistic) descriptive beliefs to Rover, or to characterize the ‘mode of presentation’ under which Rover believes the Russellian proposition <Superman, is a friend>, or something along these lines. But, since Rover is a non-linguistic creature we obviously cannot take these implicatures to have a straightforward linguistic subject matter. We cannot take them to concern, say, how Rover would linguistically express his belief or whether he would accept our report.

In contrast consider the case of Sally who is a fan of Bob Dylan and learns at time *t* that Bob Dylan was named “Robert Zimmerman.” Utterances of (5) made prior to time *t* will typically strike us as false at their time of utterance, while utterances of (5) made after *t* will strike us as true:

(5) Sally believes that Bob Dylan is Robert Zimmerman.

Suppose we try to explain these intuitions in terms of utterances of (5) generating a conversational implicature that is false before *t* and true after *t*. Now it seems that Sally does not gain any new non-linguistic descriptive beliefs about Bob Dylan when she learns he was named “Robert Zimmerman.” And it is not at all clear that Sally acquires a new ‘mode of presentation’ of Bob Dylan either.^1^ So we cannot invoke any purported implicatures concerning such things to explain our intuitions. Rather it would be natural to explain our intuitions by maintaining that utterances of (5) generate an implicature concerning Sally’s linguistic beliefs or her linguistic dispositions. It seems, then, as if the implicatures at work in (3)-(4) will have to have a different sort of subject matter from those at work in the case of (5). As noted, Berg’s allows for this while still providing a uniform account of what is going on with (3)-(5).

The second attractive feature of Berg’s account is this. On what we might call *Classical Gricean* accounts of conversational implicature, the implicature generated by an utterance in a conversational context is determined by its semantic content and that conversational context.^2^ The problem here is that, on the Millian view, utterances of (1) and (2) have precisely the same semantic content. So it seems, given a particular conversational context, utterances of (1) and (2) should generate precisely the same conversational implicatures. As we will see, Berg’s account nicely side-steps this difficulty.

Let us turn to the details of Berg’s account. Berg plausibly argues that Grice’s first Maxim of Quantity, the requirement that speakers be adequately informative for the purposes of the conversation, entails what he calls *The Principle of Implied Normalcy*:

*The Principle of Implied Normalcy*: Speakers generally conversationally implicate that the circumstances regarding whatever they are speaking of are not abnormal in any significant, unanticipated, unindicated way.

The thought here is this. Suppose you and I are talking about an *F*, *a*, and it is common knowledge that *F*s are typically *X*.^3^ You will naturally be assuming that *a* is *X*. Suppose moreover that whether or not *a* is in fact *X* is of importance and interest to you, at least given the purposes of our conversation. If *a* was not *X*, then my failing to tell you will violate the Maxim of Quantity. I will be withholding conversationally significant information from you. Because of this, if we are talking about *a* and I do not mention that *a* is not-*X*, I conversationally implicate that it is *X*.

Let us rehearse an example. Suppose I am talking about the Dean to you say “I saw the Dean today.” Typically Dean sightings are of live Deans and this will be common knowledge. Moreover we can suppose that the demise of the Dean would be of considerable interest to you. Had I seen the corpse of the Dean then I will violate the Maxim of Quantity if I do not mention this. So if I simply say “I saw the Dean today” and leave it at that, I will conversationally implicate that the Dean was alive when I saw them.

Berg then applies this principle to attitude reports. Firstly he suggests that, in general, it is normally the case that correct attitude ascriptions are acceptable verbatim to their subjects, provided those subjects understand the language in which the ascription is made and know enough about the context of utterance to resolve any ambiguities and determine the referents of any indexicals in the ascription. Suppose for example that Sally is an English-speaker and I say “Sally believes that snow is white.” If this report were correct we would normally expect Sally to accept it, to maintain when told what I had said, that I had spoken correctly. This, Berg suggests, is a common (although not exceptionless) regularity. Our attitude reports are *normally* acceptable verbatim to their subjects. More precisely:

*NormAR1*: If the subject of a report speaks the language in which the report is made then they will accept the report verbatim (after it has been adjusted to remove any ambiguities and referents have been determined for any indexicals it contains).

Because of this when I utter the sentence “Sally believes that snow is white” I implicate that Sally would accept that report verbatim. If I was aware that Sally would not accept that report verbatim, then to avoid violating the Maxim of Quantity, I would have to mention this fact.

Let us consider (1) and (2). Given *NormAR1* and the principle of implied normalcy, an utterance of (1) will standardly implicate that Lois Lane would accept (1) verbatim. And an utterance (2) will standardly implicate that Lois would accept (2) verbatim. But, of course, the first of these implicatures is true while the second is false. And it is this, Berg suggests, that explains why we hear utterances of (1) as true and utterances of (2) as false.

Berg notes that sometimes the conversational context will block such implicatures. Even if *F*s are normally *X*, in a context where we are explicitly discussing *F*s that are not-*X*, our assertions about *a* will not implicate that it is X. Likewise consider a context where we are talking about how well Superman fools Lois Lane with his Clark-Kent disguise. Here the normal presumption, that reports of Lois’s beliefs would be accepted by her verbatim, is suspended. In such a context it will be clear to everyone that Lois may not express all the beliefs she has about Superman using the name “Clark Kent” and hence that she might well not accept reports such as (2) verbatim. So my utterance of (2) in that context will not implicate that Lois would accept my report verbatim. And consequently we will not hear my utterance of (2) in that context as being false.

Berg goes on to extend his account to cover cases where we do not strictly presume the verbatim acceptability of attitude reports but nevertheless such reports generate implicatures via the principle of implied normalcy. Firstly he notes that, in cases where we do not take the subject of the report to speak the language in which the report is made, we will obviously not presume that the report is acceptable verbatim to it’s subject. However we *will* presume that a translation of the report into a language the subject understands will be acceptable to them verbatim. Thus, suppose Pierre and his audience do not think Lois Lane can speak French. When Pierre utters the sentence “Lois Lane croit que Clark Kent est un journaliste” he will not implicate that Lois would accept his report verbatim. Rather he will implicate that she would accept a translation of his report into a language she speaks. More precisely, we have the following regularity:

*NormAR2*: If the subject of the report does not speak the language in which the report is made then they will accept verbatim the result of translating the original report into their own language.

Next Berg considers cases where the idiolect spoken by the report’s subject simply does not contain the names used in the report or any reasonable translation of them. Suppose, for example, Lois Lane has not yet learned the names “Superman” and “Clark Kent” but nevertheless thinks there are two distinct individuals who have moved to town, one of whom is a flying superhero, the other of whom is a mild-mannered reporter who works in her office. If we know this, Berg suggests, my utterance of (1) or (2) would not implicate that Lois would accept my report verbatim. However we standardly associate a certain description (*the flying superhero who...*) with the name “Superman” and a certain description (*the mild-mannered reporter who*…) with the name “Clark Kent.^4^” And the following regularity holds for belief reports:

*NormAR3*: If we standardly associate certain descriptions with the name “N,” then we will normally report someone whose idiolect lacks the name “N” as believing that N is F only if they would accept verbatim the report formed from ours by replacing the occurrence of “N” with one of those descriptions.

Thus, in the circumstances we are considering, my utterance of (1) would implicate that Lois would accept verbatim a reformulation of (1) in terms of the descriptions we standardly associate with “Superman” (“Lois Lane believes the flying superhero who… is a superhero”). And my utterance of (2) would implicate that Lois would accept verbatim a reformulation of (2) in terms of the descriptions we standardly associate with “Clark Kent” (“Lois Lane believes the mild-mannered reporter… is a superhero”). In so far as we judge the first of these implicatures to be true and the second false, we will hear utterances of (1) as being true in this context and utterances of (2) as being false.

Although Berg does not discuss such cases, presumably, given *NormAR2* and *NormAR3*, if (i) the subject of our report does not speak our language and (ii) the subject’s language lacks “N” or any name that can legitimately be translated into our language as “N,” then our reporting practices conform to the following regularity:

*NormAR4*: If we standardly associate certain descriptions with the name “N,” then we will normally report someone (i) who doesn’t speak our language and (ii) whose language lacks “N” or any name that can legitimately be translated into our language as “N,” as believing that N is F only if they would accept verbatim the translation into their language of the report formed from ours by replacing the occurrence of “N” with one of those descriptions.

Berg rightly emphasizes that a single utterance may generate multiple conversational implicatures and that, indeed, it will typically generate multiple implicatures in virtue of the principle of implied normality. So one question that arises at this point is whether some weakened version of *NormAR3* holds even in cases where *NormAR1* applies. That is to say, even if Lois Lane’s lexicon contains the names “Clark Kent” and “Superman,” does my utterance of (1) implicate that she would accept verbatim some report formed by replacing the occurrence of “Superman” in (1) with one of the descriptions we standardly associate with the name “Superman?” Does my utterance of (2) implicate that she would accept verbatim some report formed by replacing the occurrence of “Clark Kent” in (2) with one of the descriptions we standardly associate with the name “Clark Kent?” And, more generally, does the following regularity hold:

*NormAR5*: If we standardly associate certain descriptions with the name “N,” then we will normally report someone as believing that N is F only if they would accept verbatim the report formed from ours by replacing the occurrence of “N” with one of those descriptions.

It is hard to see why, if our reporting practices are sensitive to a subject’s descriptive beliefs in the way captured by *NormAR3* when that subject does not understand the name used in the report, these practices would become insensitive to those beliefs when the subject learns the name. So perhaps we should allow that, in general, utterances of (1) and (2) are governed by both *NormAR1* and *NormAR5*. Perhaps we should allow that such utterances generate (at least) two conversational implicatures. At any rate, if we do so, we can explain the following phenomenon.

Consider the following scenario. Lois Lane does not realize that the mild-mannered reporter who shares her office is the same person as the caped flying superhero in the news. But she has become very confused. Somehow or other she has come to believe that the mild-mannered reporter in her office is called “Superman.” And she believes that the flying superhero in the news is called “Clark Kent.” Now consider utterances of (1) and (2) made in this context. Are these utterances intuitively correct or not? I personally find myself pulled in two different directions here. On the one hand I am tempted to count (1) as incorrect and (2) as correct, presumably because in the scenario we are considering Lois Lane would herself accept (2) but not (1). But I am also tempted to count (1) as correct but (2) as incorrect, presumably because of the descriptions we, but not Lois Lane, associate with the two names. Lois Lane might not accept an utterance of (1) verbatim, but nevertheless it still seems to get something right that (2) gets wrong. If we accept *NormAR5* then Berg’s account can explain this tension, this pull in two directions. For in the scenario we are considering utterances of (1) and utterances of (2) will each generate (at least) two distinct implicatures, one of which is true and the other false.

Berg finally considers the case of belief attributions to non-linguistic creatures. Recall that Rover responds to Superman in different ways depending upon whether Superman is dressed as a superhero or is wearing his Clark - Kent outfit. Rover wags his tail and exhibits excitement in situations of the former sort but snarls at Superman in situations of the latter sort. In these circumstances utterances (3) will strike us as true while utterances of (4) strike us as false:

(3) Rover believes that Superman is his friend,
(4) Rover believes that Clark Kent is his friend.

Obviously, however, since Rover speaks no language, we cannot explain our intuitions about (3) and (4) by appealing to any of the regularities *NormAR1*-*NormAR5* that we have considered so far. However Berg suggests that the following regularity holds in these sorts of cases:

*NormAR6*: If we standardly associate certain properties P1 with the name “N” and certain distinct properties P2 with the name “M,” and if a non-linguistic subject *x* exhibits two distinct patterns of behavior N* and M* in virtue of which we would say that they believe there are two distinct individuals, one with properties P1 and the other with properties P2, then we will normally report *x* as believing that N is the F if *x* exhibits N* behavior towards the F.

Berg’s discussion of such cases is rather brief. But I want to conclude my presentation of Berg’s account by suggesting that, if we are willing to allow ‘patterns of behavior’ to include a subject’s linguistic behavior and linguistic dispositions, we might ultimately see all of *NormAR1*-*NormAR6* as instances of a more general regularity:

*NormAR7*: If we standardly associate certain properties P1 with the name “N” and certain distinct properties P2 with the name “M,” and if a subject *x* exhibits two distinct patterns of behavior N* and M* in virtue of which we would say that they believe there are two distinct individuals, one with properties P1 and the other with properties P2, then we will normally report *x* as believing that N is the F if *x* exhibits N* behavior towards the F.

For example, we associate the property *being the bearer of* “*Superman*” with the name “Superman” and the property *being the bearer of* “*Clark Kent*” with the name “Clark Kent.”^5^ Lois Lane clearly exhibits two distinct patterns of behavior, broadly construed, S* and CK*, in virtue of which we would say she believes there are two individuals, one bearer of “Superman” and the other the bearer of “Clark Kent.” When Lois exhibits S* behavior towards the F, this includes a disposition to accept verbatim reports of the form “Lois believes that Superman is the F.” And when Lois exhibits CK* behavior towards the F, this includes a disposition to accept verbatim reports of the form “Lois believes that Clark Kent is the F.”

Obviously *NormAR7* is formulated in such general terms that, even if we accept it, it is not very helpful as it stands. But I introduce *NormAR7* to re-emphasize that Berg provides a unified overall account of what is going on in all Frege-cases. The sorts of implicatures generated in some cases will differ from those generated in others. And the regularities that ground these implicatures may vary from case to case. But all the implicatures ultimately arise from the principle of implied normalcy and, indeed, we might view *NormAR7* as further articulating what they all have in common.

## Three Objections with Responses

I now turn to considering three potential objections to Berg’s account and exploring how Berg might respond. One initial worry one might have with Berg’s account concerns whether the principle of implied normalcy can really do the work required. That principle is formulated in terms of normalcy for the circumstances regarding the *subject matter* of the conversation. It is natural to understand it as requiring speakers to report any relevant abnormalities in their subject matter; if the speaker does not indicate otherwise, her assertions about that subject matter will implicate that it is normal in those ways that are of interest and relevance to her interlocutors. However, in Berg’s account, the relevant abnormalities that the co-operative speaker is required to report do not seem to concern the subject matter of the conversation (the propositional attitude being reported) but rather our manner reporting it. The important implicatures generated by utterances of (1) and (2) are not that Lois’s belief is normal but that we are reporting it in the normal sort of way. So, one might worry, Berg cannot in fact appeal to the principle of implied normalcy to explain how these implicatures are generated.

There are two points to be made in response to this worry. Firstly, and less importantly, the letter of the principle of implied normalcy is in fact compatible with the use Berg makes of it. At any rate, the principle is formulated in terms of the ‘circumstances surrounding’ a conversation’s subject matter, and this notion seems sufficiently broad and general to include our manner of describing that subject matter (and much else besides). Obviously, however, this simply raises the question of whether the principle should really be formulated in such a broad and general way.

The second point to make is that it should, or at any rate there is a plausible similar principle that we should accept. It is undeniable that, in many contexts, a speaker’s use of one particular work or phrase rather than another can convey information, either about the speaker’s own attitudes towards her subject matter or about that subject matter itself. In particular, this can occur when we violate a normal regularity or pattern in the way words are used. And there is, of course, a very respectable body of work concerning Neo-Gricean M-Implicatures, where the use of a prolix of ‘marked’ expression generates a conversational implicature that the situation being described is abnormal or non-stereotypical.^6^ A classic example is illustrated by the contrast between:

(6) John stopped the car.
(7) John caused the car to stop.

(7) is marked in a way that (6) is not and its use implicates that John stopped the car by some abnormal means, say by driving it into a wall.

One might have two worries about appealing to M-Implicature here. Firstly M-Implicature is not take to arise as a result of the Maxim of Quantity but is taken as a distinct form of implicature in its own right. So an appeal to M-Implicature would involve a departure from Berg’s official account of how the implicatures he invokes are generated. I am not sure whether Berg would see this as a significant revision of his account. But, in any case, I take it that Berg would not be unduly concerned if his account needed such a revision.

Secondly, and more seriously, one might worry that cases such as (1) and (2) are rather different from the standard examples of M-Implicature. For (1) is neither more, nor less, prolix than (2), and it is hard to see why either (1) or (2) might count as marked. At any rate, neither (1) or (2) seems to stand to the other in the sort of relationship that (7) stands to (6). So one might worry that the Neo-Gricean theory of M-Implicature will be of no use here.

Nevertheless, I think there are some examples (first noted, I believe, by Dolf Rami) that help Berg here. If, when asked whether St. Petersburg is a beautiful city, you reply that Leningrad is indeed a beautiful city, I am likely to conclude that you lament the demise of the Soviet Union or perhaps that there has been a communist coup and the city has been renamed again. These days we normally refer to the Russian city using the name “St. Petersburg.” We expect people to use that name. And should they fail to do so we will assume there is a specific reason for this and try to interpret them accordingly. Note, conversely, that it would seem less appropriate to refer to the Russian city this way if we were talking, not about its present day condition, but rather how things were under communist rule. It would be normal say that Stalin visited Leningrad in 1950, not that he visited St. Petersburg in 1950. If you say “Stalin visited St. Petersburg in 1950” I will assume there is a specific reason for you using the name “St. Petersburg” (perhaps because you reject the legitimacy of the Soviet state) and will try to interpret you accordingly.

Again consider the names “Richard III” and “Richard of York.” These names co-refer, they both designate the same individual.^7^ Nevertheless when discussing that individual after he became king we would expect someone to use the name “Richard III” rather than “Richard of York.^8^” If you uttered the sentence “Richard of York lost the battle of Bosworth” I would be likely to assume there is a specific reason why you used this name and try to interpret you accordingly (perhaps, for example, you think that he was not the rightful king).^9^

These sorts of examples are obvious rather different from cases such as (1) and (2). But they do suggest that we are sensitive to the precise words used in an utterance, that the violation of a linguistic regularity can generate a conversational implicature that P, and hence that conforming to that regularity may generate an implicature that not-P. I am not sure best to describe what is going on in these cases. Perhaps we should say that sometimes, given a pair of co-referential names, one of the names counts as ‘marked’ or abnormal when we are discussing certain subject matters (say, the state of contemporary Russian cities), while the other counts as ‘marked’ or abnormal when we are discussing others (say, the state of Russian cities in 1950). Our normal practice when discussing a given subject matter is to use names that do not count as marked with respect to that subject matter. And so in using a name “N” I conversationally implicate that my subject matter is not one for which “N” counts as unmarked. Perhaps then the use of “Clark Kent” to describe beliefs that Lois would not self-report using “Clark Kent” should count as a marked use and the use of “Clark Kent” to describe beliefs she *would* self report using that name as unmarked. So we will naturally take an utterance of (2) to implicate that it’s subject matter is one for which the name “Clark Kent” counts as unmarked. That is to say, it will implicate that Lois would accept the report verbatim. And likewise utterances of (1) will implicate that their subject matter is one for which the name “Superman” counts as unmarked, that Lois would accept the report verbatim. A similar story might be told with respect to the other regularities in our reporting practice discussed above. So we might try to give an account of Frege-cases in terms of M-Implicature in the way noted above. Alternatively we might take the examples considered to provide an independent motivation for formulating the principle of implied normalcy in the sort of broad way Berg does. Either way, however, I think Berg has a response the first worry I raised. Given that the regularities articulated by *NormAR1-NormAR7* hold, we might appeal to either M-Implicature or to Berg's broad principle of implied normalcy to explain how utterances of (1) and (2) generate different implicatures.

The second worry I want to address concerns whether, in fact, the purported regularities articulated by *NormAR1-NormAR7* do in fact hold. I will discuss *NormAR1* here but what I have to say will apply to *NormAR2*-*NormAR7* as well. Is it *really* normal for our belief reports to be acceptable verbatim by their subjects and do we *really* presume that this is so? Berg notes, as we have seen, that we will not make this presumption in contexts where we are explicitly discussing the way the subject of a report is mistaken or fooled about the object of her belief. But I suspect the range of cases in which we do not presume verbatim acceptability is far more widespread than this. Suppose we are talking about Roman philosophy, or about the views of our colleagues. You tell me that your colleague Sally believes that Tully is morally insightful. Does this really carry the implication that Sally would accept that report verbatim? It is not at all obvious to me that my utterance does. Would we really hear my utterance as false if it turned out that Sally did not realize that “Cicero” and “Tully” co-refer and  mistakenly thought that “Tully” referred to an immoral Greek? I, for one, would not make that judgment.

And these sorts of examples can, of course, be multiplied. Suppose we are talking about great writers. You tell me that your well-read friend Mary thinks Eric Blair is the greatest writer in the English language. I doubt that we would take your utterance to be false if we discovered that Mary did not know that “Eric Blair” and “George Orwell” co-refer.

Of course, I have simply reported my own intuitions about the two cases I presented. So one obvious response would be to claim that my intuitions are idiosyncratic, that few others will share them, and so my intuitions do not undermine the claim that, save in a few abnormal contexts, most people standardly presume the verbatim acceptability of attitude reports. However this response is ultimately besides the point. The cases I presented suggest that I do not, in general, standardly presume the verbatim acceptability of the attitude reports I hear. Nevertheless, in many contexts, I will hear an utterance of (1) as in some sense true or correct but an utterance of (2) as false or incorrect. Whatever the reason for this, it cannot simply be because I standardly presume the verbatim acceptability of attitude reports. Some other explanation of my intuitions is required. And, of course, one might well suppose that whatever this explanation might turn out to be, it is likely to apply across the board. It is likely to be the explanation, not merely of my own intuitions about the relevant utterances of (1) and (2), but the intuitions of others as well.

There is, however, a better response available to Berg. Recall that in the Sally case I presented above we were talking about Roman philosophy, or about the views of our colleagues. Your primary interest here was simply to inform me of what Sally thought about Cicero/Tully, of her opinion of his moral philosophy. In other words, for the purpose of our conversation, your primary aim was to inform me that she believed a certain Russellian proposition. Once you had conveyed the relevant information about Sally’s attitude towards Cicero/Tully you had finished your communicative task. No further information, for example how Sally would report her own attitudes, was necessary given the purpose of our conversation. Likewise, in the Mary-case, the only information that is relevant to the conversation is that your well-read friend’s opinion of George Orwell/Eric Blair. The fact she thinks so highly of his writings is a mark in their favor. How exactly she might express her attitudes is of no direct relevance, given the purpose of our conversation.

These situations of course contrast with cases where we are interested in predicting or explaining someone’s verbal or non-verbal behavior. Suppose Lois Lane smiles when she sees Superman/Clark Kent in his Superman outfit and frowns when she sees him dressed as a mild-mannered reporter. Simply being informed that Lois believes a Russellian proposition to the effect that Superman/Clark Kent is heroic and that she believes a Russellian proposition to the effect that Superman/Clark Kent is insipid will not adequately explain her behavior. We need our explanation to account for the fact that Lois behaves differently towards the same individual in different circumstances.

I suggest that we are inclined to hear attitude reports as conveying a *de dicto* content in contexts where we are interested in explaining or predicting the behavior of the report’s subject, or at least where we recognize the potential need to do so. It is in these contexts that we make the sorts of normalcy presumptions articulated in *NormAR1-NormAR7*. And so it is in these contexts that utterances of (1) and (2) generate the sorts of implicatures that Berg discusses. While departing from the letter of Berg’s account I think this suggested amendment stays true to its spirit. And, as we shall see, the amendment might allow him to avoid a further worry.

The third and final objection to Berg’s account that I want to discuss is this. We might well wonder precisely *why* we should make the sorts of presumptions of normalcy we have been discussing and why the patterns and regularities these presumptions concern should hold at all. Why, in context where we might be interested in explaining Lois Lane’s behavior, is it normal to report her beliefs in a manner that would be acceptable to her verbatim? Why, in such a context, would it be normal to report her beliefs using a name we standardly associate with descriptions such that Lois would accept the report formed by replacing the occurrence of that name in our report with those descriptions? No doubt, once these regularities were in place, the conversational implicatures they gave rise to would reinforce and perpetuate them. But how did they arise in the first place?

It might be, of course, that these patterns are simply a brute fact. Perhaps somehow or other we started making reports which happened to exhibit these regularities. And, once this pattern was established and (perhaps implicitly) recognized by speakers, the conversational principle of implied normalcy (or mechanism of M-Implicature) guaranteed that cooperative speakers would conform to that pattern and thereby reinforce and perpetuate it. In the end perhaps this is all we will be able to say. Nevertheless I think there is obviously something unsatisfactory about taking the relevant patterns as simply a brute fact in this way. So I want to explore a more satisfactory explanation.

Here are two rather speculative suggestions that I want to explore. The first suggestion takes up an idea developed by among others Gordon (1986), Goldman (2006), and Currie and Ravenscroft (2002), the idea that mindreading involves simulation. On this view, when we try to interpret another individual we in some sense try to cognitively simulate their situation. We take our cognitive mechanisms ‘off-line,’ as it were, we simulate what we take to be their beliefs and desires, and we try to determine what behavior those beliefs and desires would give rise to. This view is not uncontroversial, to say the least. Nevertheless I offer my first suggestion to those sympathetic to simulation accounts of mindreading.

Suppose that I realize, but you do not, that Lois believes that Superman is in the next room. Lois Lane runs towards the next room. You ask me why she is doing this. I need to offer an explanation of her behavior so I need to engage in mindreading; I need to simulate her cognitive state. In doing this I will simulate her belief in the Russellian proposition that Superman is in the next room. But I will also simulate any other descriptive beliefs I take her to have. I will then utter a belief report that articulates my simulation of Lois’s cognitive state. In effect I utter a report whose complement sentence *S* is the sentence I think, given my simulation of her cognitive state, Lois would utter herself to explain her behavior. In this way we can see why, in contexts where we are interested in explaining someone’s behavior, it would be normal for our reports to be ones that were acceptable verbatim to their subjects, or at least to be ones that we supposed to be verbatim acceptable. Likewise, when we consider agents who do not speak our language, we will attempt to simulate what we take to be their cognitive state and their beliefs. And the complement sentence of our report will be one that, were we to really have the beliefs we simulate, we would use in a self-report. Where the subject of our report speaks a foreign language our complement sentence will normally be a translation of the foreign sentence the subject would themselves use in a self-report. And where the subject is a non-linguistic agent our complement sentence will be one we would use, were we to have the descriptive beliefs we simulate the agent as having. 

My second suggestion is similar and also invokes the notion of simulation, but in a far weaker sense, and it is compatible with alternative accounts of mindreading. I suggest that, in the first instance, we tend to defer to an agent when it comes to explaining their behavior. We take the best explanation of their behavior to be the one which they themselves would offer us. And, when we try to explain the behavior of someone else, in a certain sense we try to speak for them. We try to say what (we think) they would say in order to explain their behavior, given the overall cognitive state we take them to be in. In particular we aim for the complement sentence of our report to be the sentence that the subject of the report would themselves offer as an explanation of their behavior. Once again we get the same results. It would be a regularity that the belief reports we make to explain the behavior of others would be verbatim acceptable to their subjects, or at least we would take them to be so. When we consider agents who do not speak our language the situation is obviously rather different. But I suggest a similar tendency to deference occurs and we utter reports that we presume the agent would use, were they to speak our language. Once again, where the subject of our report speaks a foreign language our complement sentence will normally be a translation of the foreign sentence the subject would themselves use in a self-report. And where the subject is a non-linguistic agent our complement sentence will be one we would use, were we to have the descriptive beliefs we take the non-linguistic agent to have. 

## Footnotes

1 In so far as we can make sense of the idea of a ‘mode of presentation’ or ‘way of thinking about’ an object, it is hard to see how Sally gains a new mode of presentation or way of thinking about Bob Dylan. Rather she surely simply associates a new name with the mode of presentation or way of thinking about Dylan that she already has.

2 See, for example, Grice (1975) p. 31.

3 Strictly speaking perhaps we need to assume, not this, but rather that it is be common knowledge that people *take F*s to generally be *X*. Since this complication will not matter in what follows, I will ignore it for the moment.

4 Note that associating a property or description with a name is obviously not the same as taking the name to have that property or satisfy that description. Rather it is to believe the bearer of the name has that property or satisfies that description.

5 Remember, associating a property with a name is *not* the same as taking the name to have that property. Rather it is to believe the bearer of the name has that property.

6 See, for example, Levinson 2000, and Huang 2000 and 2007.

7 Despite superficial appearances I think both expressions are best understood as semantically simple proper names.

8 Perhaps when discussing that individual’s early life we would also expect the converse to be true, although to a lesser extent, I think.

9 Note that in both these cases the speaker might go on to ‘cancel’ the conclusion I draw. In the first case you might add “Sorry, I grew up during the Cold War and just cannot get out of the habit of calling the city that name.” In the second case you might add “Not that I deny he was rightfully king.”
